# Glycan-Based Electrochemical Biosensors: Promising Tools for the Detection of Infectious Diseases and Cancer Biomarkers

**DOI:** 10.3390/molecules27238533

**Published:** 2022-12-03

**Authors:** Danilo Echeverri, Jahir Orozco

**Affiliations:** Max Planck Tandem Group in Nanobioengineering, Institute of Chemistry, Faculty of Natural and Exact Sciences, University of Antioquia, Complejo Ruta N, Calle 67 N°52–20, Medellin 050010, Colombia

**Keywords:** glycan, electrochemical glycobiosensor, infectious disease, cancer biomarker

## Abstract

Glycan-based electrochemical biosensors are emerging as analytical tools for determining multiple molecular targets relevant to diagnosing infectious diseases and detecting cancer biomarkers. These biosensors allow for the detection of target analytes at ultra-low concentrations, which is mandatory for early disease diagnosis. Nanostructure-decorated platforms have been demonstrated to enhance the analytical performance of electrochemical biosensors. In addition, glycans anchored to electrode platforms as bioreceptors exhibit high specificity toward biomarker detection. Both attributes offer a synergy that allows ultrasensitive detection of molecular targets of clinical interest. In this context, we review recent advances in electrochemical glycobiosensors for detecting infectious diseases and cancer biomarkers focused on colorectal cancer. We also describe general aspects of structural glycobiology, definitions, and classification of electrochemical biosensors and discuss relevant works on electrochemical glycobiosensors in the last ten years. Finally, we summarize the advances in electrochemical glycobiosensors and comment on some challenges and limitations needed to advance toward real clinical applications of these devices.

## 1. Introduction

Glycan-based electrochemical biosensors are analytical devices that use glycans as biorecognition elements immobilized on electrode surfaces to detect a target molecule electrochemically. Furthermore, glycan-based biosensors include biosensing platforms for detecting glycan-based biomarkers [1]. Electrochemical biosensors offer a valuable alternative for biomarker detection with high specificity, sensitivity, low cost, and the possibility of implementation in decentralized settings. In addition, they can be user-friendly, portable, amenable to miniaturization, and cost-affordable [2,3].

Glycans are carbohydrate molecules in free form or attached to other molecules such as proteins and lipids [4]. Glycans play a fundamental biological role because they participate in cell signaling and cell–cell adhesion, provide specific receptors for microorganisms, toxins, or antibodies, and regulate protein functions glycosylation dependently [5,6]. In addition, glycans are involved in cell growth and development, host–pathogen interactions and the progress of infections, immune recognition/response, tumor growth, and metastasis [5,6,7]. All these characteristics make glycans useful for the diagnosis/prognosis of diseases [8]. For example, glycans can be used as bioreceptors in sensing platforms for the detection of infectious diseases and as cancer biomarkers, including glycolipids such as glycosylphosphatidylinositol (GPI) for the diagnosis of parasites [9,10] and glycoproteins such as the carcinoembryonic antigen (CEA) and the carbohydrate antigen (CA 19-9) as biomarkers for the diagnosis of colon cancer [11,12]. In addition, other glycoproteins, such as the β-1,4-galactosyltransferase-V, have been reported as novel biomarkers of colon cancer [13,14,15].

Currently, the clinical diagnosis of some infectious diseases and cancer uses serological tests for biomarkers detection, including immunoassays [16,17] and chromatographic-based methods [18,19]. However, these methods have limitations, including laborious experimental procedures, limited multiplexing options, the need for sophisticated and centralized laboratory equipment, and skilled personnel [20,21]. In this context, glycan-based biosensors offer an alternative for biomarker detection in the clinical diagnosis of diseases, with high specificity, sensitivity, rapid response, low cost, and opportunity for miniaturization and portability [1,22]. These attributes make glycan-based biosensors ideal candidates for device-based disease diagnosis close to the patient, for example, in low- and middle-income settings with limited resources. Furthermore, they offer the advantage of short assay time, high portability, and multiplexing ability, enabling the possibility of implementation at the point-of-care (POC) [23].

This work aimed to review glycan-based electrochemical biosensors for detecting infectious diseases and cancer biomarkers. First, relevant aspects of structural glycobiology and the concepts of electrochemical glycobiosensors are briefly described. In addition, representative electrochemical glycobiosensors for detecting infectious diseases and cancer biomarkers reported in the last ten years are discussed to provide a more comprehensive background. This work contributes to the state of the art of glycan-based biosensors, especially toward their application in the clinical diagnosis of infectious diseases and detection of cancer biomarkers, with a particular focus on colorectal cancer and emphasis on electrochemical impedance spectroscopy (EIS) and electrochemical capacitive spectroscopy (ECS) transduction.

## 2. Structural Glycobiology

Glycans are carbohydrate compounds (polyhydroxy -aldehydes or -ketones) in their free form or monosaccharide units covalently linked by glycosidic bonds in the form of oligosaccharides and polysaccharides. In addition, glycoconjugate refers generically to any carbohydrate or assembly of carbohydrates covalently attached to another molecule (mainly proteins and lipids) [24].

Both eukaryotic and prokaryotic cells synthesize glycoconjugates, including glycoproteins, GPI-anchored glycoproteins, proteoglycans, and glycosphingolipids [25]. Glycoproteins are the main class of glycoconjugates [26] and consist of polypeptides that have glycans covalently linked to asparagine and serine/threonine residues (N-glycans and O-glycans, respectively) and through C-mannosylation, where a covalent bond between carbon one of the mannose and carbon two of the indole ring of tryptophan is formed [5].

GPIs are glycolipids with a conserved core structure of phosphatidylinositol-lipid linked to no-acetylated N-acetyl glucosamine and three mannose residues followed by an ethanolamine [27]. Generally, GPIs have the function of anchoring proteins on the cell membrane in eukaryotes and archaea; additionally, non-protein (linked free GPIs) are abundant on the surface of several protozoan parasites, such as *Trypanosoma brucei*, *Plasmodium falciparum*, and *Toxoplasma gondii* [5]. In addition, GPI-anchored proteins participate in cell signaling and adhesion and are related to health and disease processes [28].

Proteoglycans are macromolecules that consist of a protein backbone to which glycosaminoglycan chains (GAGs) and N- and or O-linked oligosaccharides are covalently attached. GAGs are linear and negatively charged polysaccharides composed of repeating disaccharides of acetylated hexosamines (N-acetyl-galactosamine or N-acetyl-glucosamine) and mainly by uronic acids (d-glucuronic acid or l-iduronic acid) sulfonated at various positions [29]. Ceramides (Cer) are the core structure of sphingolipids composed of a fatty acid linked by an amide bond to the unsaturated amino alcohol sphingosine. In addition, ceramides may have glycans attached and other polar phosphate-containing head groups [30]. Proteoglycans and sphingolipids are involved in cell signaling and, therefore, are related to the development of different human diseases [29,31,32,33].

### 2.1. Biological Functions of Glycans

The chemical diversity and structural complexity of glycans imply that they have diverse biological functions [5]. For example, glycans participate in cell–cell adhesion and cell signaling, provide specific receptors for microorganisms, toxins, or antibodies, and modulate protein functions in a glycosylation-dependent manner [7]. Furthermore, they participate in the folding of many proteins and protein trafficking, differentiate blood groups, participate in different signaling pathways, and play a fundamental role in the infectivity process of many pathogenic bacteria and viruses [6].

### 2.2. Principles of Glycan Recognition

Glycans can interact with different types of receptors, including biomolecules such as proteins (antibodies and lectins) [13,14]; aptamers, which are short, single-stranded oligonucleotides (DNA or RNA) [15]; and other smaller synthetic receptors such as boronic acids [16]. Overall, lectin–glycan interactions and antibody– and aptamer–carbohydrate complexes are held together by hydrogen bonds, CH-π, Van der Waals, and electrostatic interactions [17]. For example, it has been reported that antibodies can interact with glycans via tryptophan residues of antibodies and OH groups of glycans mediated by OH-π and CH-π interactions. In addition, hydrogen bonds among mainly polarized residues of aspartate, histidine, lysine, and threonine of antibodies with the mannose of the glycans also contribute to their recognition specificities [34]. On the other hand, in the case of nucleic acids, highly polar glycans can interact with oligonucleotides by stacking on cytosine–guanine base pairs through CH-π interactions. In addition, when hydrophobic contacts are available, apolar glycans can interact with loop DNA bases through hydrogen bonding [35]. In summary, the predominant type of interaction will depend on the structure of the glycan and the receptor to which it binds. These interactions between glycans and other biomolecules establish the principle of biomolecular recognition events on sensing surfaces that are converted into a readout signal by the transducer. For this reason, it is mandatory to know the type of biomolecular interactions between the bioreceptor and analyte to design the sensing surface.

### 2.3. Glycan-Based Biomarkers for Diagnosis of Diseases

According to the International Program on Chemical Safety, a biomarker is any substance, structure, or process that can be measured in the body or its products and influence or predict the incidence of outcome or disease [36]. As mentioned above, glycans are biomolecules regulating human physiology and pathology, including cell signal transduction and microbial infections. Therefore, diagnosing infectious diseases and cancer often uses glycan-based biomarkers [8].

#### 2.3.1. Infectious Diseases

Identifying pathogens that cause infections, such as viruses, bacteria, fungi, and protozoa, is possible by detecting glycan-based biomarkers. Glycans are present on the outermost surface of viruses, mainly in glycoproteins [37]. Therefore, detecting structural glycoproteins on viruses, or quantifying titers of antibodies in the host against viral antigenic glycoproteins, allows the diagnosis of infections by viruses [37]. Some examples of virus determination based on glycoprotein detection are coronaviruses by detecting Spike glycoprotein [38,39], E1 and E2 glycoproteins in hepatitis C and Chikungunya viruses, and NS1 glycoprotein in dengue virus [37]. Like viruses, an array of glycans covers the bacteria cells, comprising their cell wall [24]. In addition, there are sugars with a limited expression on pathogenic bacteria; for example, *Neisseria meningitides* utilizes 2,4-diacetamido-2,4,6-trideoxyhexose, *Pseudomonas aeruginosa* installs N-acetylfucosamine residues, and *Bacteroides fragilis* appends 2-acetamido-4-amino-2,4,6-trideoxy-galactose into its cell surface polysaccharides. These are specific sugars of pathogenic bacteria and are promising biomarkers for their detection in clinical diagnosis [40]. Moreover, pathogenic fungi determination is also possible by detecting glycan on the cell surface. Some examples of pathogen fungi diagnosis based on glycan-type antigens include the detection of the antigen mannan in fungi of the genus *Candida* and *Cryptococcus* and galactomannan in *Aspergillus* [41].

On the other hand, an alternative for infectious disease diagnosis is glycan microarrays. Glycan microarrays are arrangements of multiple glycans, or glycoconjugates, immobilized onto a solid phase platform for screening with glycan-binding proteins (GBPs, also known as lectins), antibodies, bacteria, viruses, and other microorganisms. The GBPs or microorganisms are either directly fluorescently labeled or labeled with a tag such as biotin that can be indirectly detected [42]. Glycan arrays are a tool to elucidate carbohydrate interactions with different GBPs, including soluble proteins such as immune toxins, lectins, and microbial and mammalian surface receptors [43]. Furthermore, serum antibodies and human GBPs directed against cell surface glycans have been applied for the detection of diverse pathogens, including protozoa such as *Toxoplasma gondii* and *Plasmodium falciparum*, and for a wide range of pathogenic viruses and bacteria such as influenza viruses, human immunodeficiency virus (HIV), *Salmonella* and *Burkholderia pseudomallei*, respectively [43,44].

#### 2.3.2. Cancer Biomarkers

Glycans can be used as biomarkers in cancer since they have different biological functions, especially in cell signaling pathways and post-translational protein modifications, which are related to the development and progression of this disease [45]. In this context, glycoproteins such as CEA and CA 19-9 have been used conventionally as biomarkers for cancer diagnosis, especially colon cancer [46,47,48]. CEA is a GPI-cell surface-anchored glycoprotein whose specialized sialofucosylated glycoforms serve as functional colon carcinoma L-selectin and E-selectin ligands, which may be critical to the metastatic dissemination of colon carcinoma cells [49]. CA 19-9 antigen is a tetrasaccharide carbohydrate termed sialyl Lewis-a, synthesized by gastrointestinal epithelium and overexpressed in colorectal cancer [50]. In addition, other glycoproteins, such as sialic-acid-containing glycoproteins are involved in cancer initiation, progression, and metastasis and are used as biomarkers for the disease diagnosis/prognosis [51,52,53].

Other biomarkers for cancer diagnosis related to glycan-type molecules are the enzymes involved in glycosylation and their reaction products. Glycosylation is the enzymatic process that produces glycosidic linkages of saccharides to other saccharides, proteins, or lipids [45]. A large family of enzymes called glycosyltransferases synthesizes the carbohydrate motifs of glycoconjugates. These enzymes catalyze glycosidic bond formation using sugar donors containing a nucleoside phosphate or a lipid phosphate leaving group [54,55]. Alterations in protein glycosylation are among the main molecular events accompanying oncogenic transformations in the gastric and colorectal tracts [56]. In addition, there is a report of increased glycosphingolipid levels due to aberrant glycosylation and metabolism in colorectal cancer [57]. It is relevant to emphasize that β-1,4-galactosyltransferase-V (β-1,4-Galt-V) catalyzes the glycosylation of glucosylceramide and the N-acetylglucosamine β-1-6 mannose group of the highly branched N-glycans, which are overexpressed in colorectal tumor cells [58]. This evidence indicates that β-1,4-GalT-V may serve as a diagnostic biomarker for the progression of human colorectal cancer [13].

## 3. Electrochemical Biosensors for Biomarker Detection

Electrochemical biosensing enables the detection of different analytes with high sensitivity; the equipment is simple, affordable, and amenable to miniaturization; and the electrode surface chemistry can adapt to specific applications [22,59]. Electrochemical biosensors use several electroanalytical techniques, including voltammetric techniques—such as cyclic voltammetry (CV), differential pulse voltammetry (DPV), square wave voltammetry (SWV), and amperometry—as well as potentiometric, conductometric, and spectroscopic techniques such as EIS and ECS [22,60,61]. Regarding analytical performance, EIS- and ECS-based glycan biosensors are highly sensitive compared with voltammetric and amperometric biosensors. Therefore, they are promising techniques for developing devices for detecting analytes at ultralow concentrations [62].

### 3.1. Definitions and Classification of Electrochemical Biosensors

**Figure 1:**
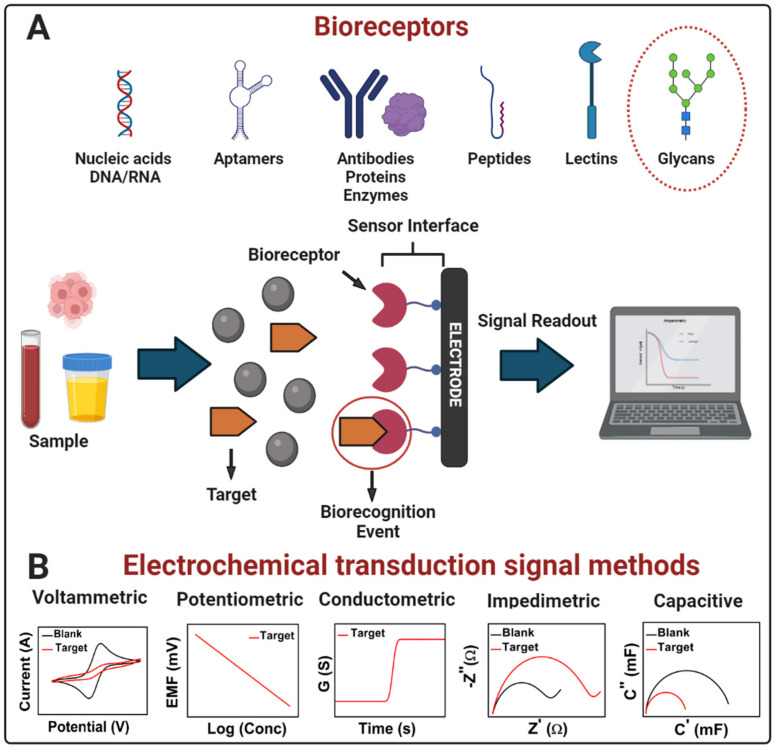
(**A**) Scheme of a generic electrochemical biosensor. A bioreceptor (nucleic acids, aptamers, antibodies, proteins, enzymes, peptides, lectins, glycans, etc.) attached to the electrode surface recruits the molecular target (analyte present in a sample) onto the sensor interface by an affinity reaction. After the bioreceptor binding with the target (biorecognition event), the transducer converts the binding event into a measurable signal proportional to the concentration of the target (signal readout). (**B**) Electrochemical transduction signal methods in biosensors: voltammetric, potentiometric, conductometric, impedimetric, and capacitive. Adapted from [22] with permission. Copyright Elsevier 2022.

An electrochemical biosensor is a self-contained integrated device that can provide specific quantitative or semi-quantitative analytical information of molecular recognition events into an analytically valuable signal using a biological recognition element (bioreceptor) that is retained in direct spatial contact with an electrochemical transduction element (Figure 1A) [47,48]. The main function of the transducer is to convert a molecular biorecognition event into a measurable signal proportional to the analyte concentration [49]. According to the type of bioreceptor, there are biosensors based mainly on whole cells, enzymes, antigens, antibodies, nucleic acids, aptamers, lectins, and glycans [49,50,51]. The bioreceptor is the element that confers specificity and selectivity to the biosensor, which are relevant characteristics of these devices, and refers to the ability to detect a specific analyte in a mixture that contains interferences [50] and differentiate the target from homologous counterparts.

Other characteristics of biosensors are sensitivity, linear range, reproducibility, and stability. Sensitivity is the slope of the calibration curve and is related to the limit of detection (LOD). The LOD is the lowest concentration of an analyte in a sample that can be detected, with reasonable certainty, for a given analytical procedure [63]. The linear range is the concentration range over which the signal output is directly proportional to the concentration of the analyte and is often correlated with a straight line [64]. Reproducibility is the closeness and agreement between independent results obtained with the same method on identical test material but under different conditions (different operators, apparatus, laboratories, or time intervals) [63]. Finally, stability is the degree of biosensor susceptibility to ambient disturbances. One way to assess the stability is by continuously or sequentially performing biosensor exposure to analyte solution or by measuring the change in the baseline or sensitivity over a fixed period [65,66]. These characteristics make electrochemical biosensors affordable, accurate, rapid, and sensitive analytical platforms for detecting multiple disease biomarkers [67].

A convenient classification of electrochemical biosensors is according to the mode of signal transduction, as mentioned in Section 3.1 and Figure 1B [22,68]. Moreover, another classification of electrochemical biosensors is according to the bioreceptor type that recognizes the analyte [68]. Therefore, biosensors based on antibodies, or fragments of these, are affinity biosensors (immunosensors); enzyme-based biosensors use enzymes as bioreceptors; genosensors use nucleic acids; aptasensors use aptamers; glycobiosensors use lectins or glycans; and cytosensors whole cells [1,22]. Since this work focused on developing electrochemical biosensors, and we will tackle the glycan-based biosensors in Section 2.3, the following classification zooms in according to the mode of signal transduction.

In summary, electroanalytical methods allow the precise determination of multiple analytes with high sensitivity and fast response. Table 1 helps sort out electrochemical glycobiosensors by comparing electroanalytical methods based on analytical performance and the detection principle.

### 3.2. Characterization of Electrochemical Glycobiosensors

The electrode surface of electrochemical glycobiosensors is usually characterized in terms of surface chemistry, morphology, and electrochemical performance. Atomic force microscopy (AFM) is a technique used to characterize the electrode surface morphology in glycobiosensors; it can operate in multiple modes, such as electrochemical AFM, which enables the analysis of electrochemical reactions occurring at the electrode [70]. Morphology and surface chemical composition are characterized via scanning electron microscopy (SEM) coupled with energy-dispersive X-ray spectroscopy (EDX) [71,72]. Furthermore, infrared (FT-IR) and X-ray photoelectron spectroscopic (XPS) techniques are used to analyze surface chemical composition [73]. In addition, thermogravimetric analysis (TGA) is applied to analyze the materials used to modify the electrode surface and implies studying sample mass change under programmed conditions. Therefore, TGA is mainly used to analyze certain thermal events, such as absorption, adsorption, desorption, vaporization, sublimation, decomposition, oxidation, and reduction [74].

Similarly, differential scanning calorimetry (DSC) measures the amount of energy absorbed or released by the sample when it is heated or cooled. TGA is a versatile technique used to study the self-assembly of supramolecular nanostructures such as glycopolymers, latent heat of melting, denaturalization temperatures, and compositional analysis [75]. On the other hand, the optical properties are mainly characterized via ultraviolet-visible (UV-vis) spectroscopy and photoluminescence (PL) techniques [76]. Finally, electrochemical performance is characterized via voltammetric techniques such as CV and DPV and spectroscopic techniques such as EIS and ECS [77,78], as commented on in Section 3.2. The affinity constants of glycan-based biorecognition elements can be determined using the Biacore system [79]. Biacore uses surface plasmon resonance (SPR) as a label-free detection technique to monitor the interaction between biomolecules in real-time. The biorecognition molecule is immobilized on a sensor chip’s surface. At the same time, the sample containing its ligand is injected over the surface at a constant flow rate through a microfluidic channel system. The changes in mass concentrations at the surface of the sensor chip due to molecule association/dissociation are measured as an SPR response and displayed as a time function [80]. Table 2 shows the most common characterization techniques of electrochemical glycobiosensors.

### 3.3. Nanostructured Electrochemical Glycobiosensors

Nanostructured biosensors are analytical devices that integrate nano- and bio-materials platforms for trace detection of biomolecules or chemical analytes [81]. Due to their size-dependent properties, such as a large surface area with improved conductivity and reactivity, nanomaterials are used for developing highly sensitive biosensors [82]. As a result, a wide range of nanomaterials has been incorporated onto the electrode surface to improve the biosensor analytical performance, such as carbon-based nanomaterials, noble metals, metal oxides, metal chalcogenides, magnetic nanoparticles, and conductive polymers, among others [83].

Likewise, nanomaterials are similar in size to most biological entities such as proteins, nucleic acids, lipids, cells, viruses, glycans, etc., making them ideal interfaces between these entities and signal transduction surfaces as those used in biosensors [84,85,86,87]. Furthermore, stability, biocompatibility, and the advantage of modulating the nanomaterial’s surface chemistry make them suitable for conjugating multiple chemical species and biomolecules [83]. One general advantage of all nanomaterials is the high specific surface area that enables a high surface loading of biorecognition elements on the electrode surface and their resultant improved electron transfer and electrocatalytic activity ability [83,88]. Their combination with suitable bioreceptors such as glycans could originate synergistic effects eliciting unforeseen benefits [89]. Therefore, an essential issue for nanobiosensor development is the size, structure, chemical composition, shape, and nanomaterial’s surface modification [87].

#### 3.3.1. Synthesis of Nanomaterials and Surface Biofunctionalization

There are different methods to synthesize nanomaterials depending on their type and nature. In summary, the two main methods to synthesize nanomaterials are “top-down” and “bottom-up” approaches [88]. In the top-down approach, the nanomaterial synthesis uses the size reduction from bulk materials down to the nanoscale. Unlike the top-down method, the bottom-up synthesis of nanomaterials consists of obtaining nanostructures from elementary-level building blocks of atomic or molecular size [88]. The most common methods used to synthesize nanomaterials are the chemical vapor deposition method, thermal decomposition, hydrothermal synthesis, solvothermal method, pulsed laser ablation, templating method, combustion method, microwave synthesis, gas phase method, and conventional sol–gel method [88].

As mentioned above, glycan-based biorecognition elements confer specificity and selectivity to glycobiosensor devices, recognizing the target analyte and binding it to the sensor surface for transduction [66]. Nanomaterials are supporting platforms for glycan-based bioreceptors attachment by physical and chemical methods [90] and enhancing analytical performance [91]. Such bioreceptors are immobilized to the nanomaterials by physical methods without chemical bond formation through physical entrapment, microencapsulation, adsorption, and sol–gel techniques [92]. Unlike physical methods, chemical approaches form covalent bonds in the presence of two mutually reactive chemical groups from the bioreceptors and the substrate surface [90,93]. One of the more common approaches involves amide bond formation in the presence of carboxylic acids (-COOH) and primary amines (-NH_2_). This approach requires activation of -COOH with 1-ethyl-3-(3-dimethylaminopropyl) carbodiimide (EDC) and N-hydroxysuccinimide (NHS) or sulfo-NHS, producing esters, which react with primary amines to form amides. Another approach couples the bioreceptor via the free thiols (-SH), which react stoichiometrically with maleimides through a Michael addition reaction [93]. The glycans also can be functionalized with thiols and disulfide-bearing linkers for the direct formation of self-assembled monolayers (SAMs) on metallic surfaces, i.e., gold. Alternatively, some linkers can be pre-assembled on the transducer surface for subsequent attachment of glycans via reactive terminal groups [94,95].

Bioreceptors, like glycans, are also immobilized onto conducting polymers with redox properties, such as polystyrene sulfonate, polyvinyl ferrocene, polythiophene, polyaniline, and quinone polymers, which results in an enhancement of the biosensor electrochemical performance [96]. Furthermore, there are immobilization strategies based on affinity interactions, such as the biotin–avidin interaction or immobilizing binding proteins such as Protein A or G onto the electrode surface, followed by the subsequent capture of antibodies and blocking of the nonspecific adsorption sites steps with bovine serum albumin (BSA), casein, or other blocking agents [90,93,97,98,99,100].

#### 3.3.2. Operation Modes of Electrochemical Nanobiosensors

**Figure 2:**
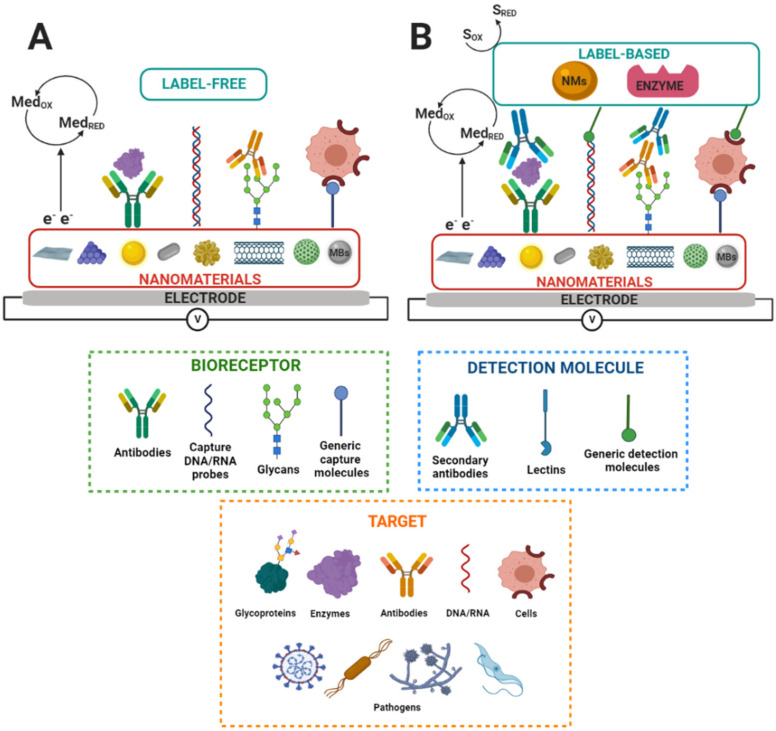
Scheme of nanostructured electrochemical biosensors. S_RED_ and S_OX_ and Med_RED_ and Med_OX_ indicate substrate reduction and oxidation and mediator reduction and oxidation, respectively. MBs indicate magnetic beads. (**A**) Label-free and (**B**) label-based nanobiosensors set up. Adapted from [83].

Electrochemical biosensors that integrate nanostructures and suitable biorecognition elements on the electrode surface are highly sensitive and specific. These features enable the detection and quantification of disease biomarkers at ultra-low concentrations, which is a requisite for the early diagnosis of diseases [88,101]. On the other hand, regarding the detection of molecular biorecognition events on the electrode surfaces, various labeling strategies are used to amplify the detection signal on electrochemical biosensors. Label-based approaches can involve avidin–biotin conjugation with redox enzymes, covalent attachment, intercalation, or electrostatic interaction of small molecules, particles, or ions with the biorecognition elements responsible for generating the electrochemical signal. In contrast, label-free biosensors directly transduce a molecular binding event into a physically measurable quantity, i.e., without needing an additional antibody, enzymatic, fluorescent, or electroactive label, or any other amplification strategy, to provide a response that is proportional to the concentration of bound molecules [102]. Label-free electrochemical biosensors measure interfacial electrical property changes, such as charge transfer resistance or electrochemical capacitance, through the EIS or the ECS techniques and by measuring current changes via CV, DPV, or SWV [22,77]. Figure 2 shows a label-free and label-based nanobiosensors setup.

In summary, electrochemical nanobiosensors based on glycans offer exceptional attributes for disease diagnosis, such as being affordable, sensitive, specific, user-friendly, portable, rapid, robust, simple to construct, equipment-free, and deliverable to all people in need [2,103]. In addition, nanostructured electrodes provide a large surface area to immobilize a high load of bioreceptors on the electrode surface and enhance the electron transfer and electrocatalytic activity ability, resulting in biosensors of enhanced analytical performance [82].

#### 3.3.3. Glycans as Biorecognition Elements in Electrochemical Biosensors

A myriad of bioreceptors, such as antibodies, nucleic acids, aptamers, peptides, enzymes, etc., have been used in biosensors as biorecognition elements for detecting multiple biomarkers [68,104,105,106]. However, the application of glycans as bioreceptors is less explored in biosensor platforms to develop new diagnosis/prognosis tests [107].

The most common types of electrochemical glycobiosensors use lectins as selective biorecognition elements. Lectins are natural proteins that recognize and reversibly bind to specific free carbohydrates and terminal groups on glycans of glycoconjugates [59,67,108,109]. Lectin-based biosensors have been used for detecting CEA, P-glycoprotein, prostate-specific antigen (PSA), and viruses [67,110,111].

On the other hand, there are some applications where glycans are attached to the electrode surface as biorecognition elements [62]. In two of these applications, a glycan sialyllactose was immobilized on SAM-modified gold electrodes via amine coupling to detect proteins present on the envelope of influenza viruses [112,113]. Similarly, another application uses a mannose glycan immobilized on SAM-modified gold electrodes for bacteria detection [114]. Furthermore, there is a report for the immobilization of Tn antigen (N-galactosamine attached to serine) on SAM-modified gold electrodes for binding a tumor-associated antibody [115]. In addition, there are reports describing glycans immobilization with a built-in redox center. In these works, glycans were attached to quinone moieties and applied to detect intact bacterial and cancerous cells using graphene-modified electrodes [116,117,118].

In summary, glycan immobilization on the electrode surface depends on the glycan structure and the electrode surface chemistry. Glycans specifically recognize molecular targets and confine them on the electrode surface. The biorecognition molecular event changes the interfacial electrical properties, and the analyte concentration correlates with the changes in electrical properties measured by electrochemical techniques.

The bioreceptor affinity is inversely related to the dissociation constant K_D_. It describes the binding strength between a bioreceptor, such as a lectin or an antibody, and its ligand [119]. K_D_ is in the nM–mM range for lectin–glycan interactions and pM-nM for oligonucleotide hybridization and antibody–antigen interactions [120,121,122,123]. Lectins have multivalence to recognize glycans, allowing significant affinity amplification and even reaching the subnanomolar range [121]. In addition, engineered glycomaterials allow for overcoming affinity and selectivity challenges in glycan-based molecular binding events achieving affinity in the picomolar range [124]. For this reason, glycans are promising bioreceptors for electrochemical biosensors, as mentioned.

## 4. Electrochemical Glycobiosensors for Infectious Disease Diagnosis and Cancer Biomarker Detection

Regarding different applications of glycobiosensors, there are some reports in the literature, mainly for the clinical diagnosis of infectious diseases caused by pathogens (such as viruses and bacteria) and the detection of cancer biomarkers. Therefore, below, this section describes representative works on electrochemical glycan-based biosensors for pathogens and colorectal cancer biomarkers in the last ten years.

### 4.1. Glycobiosensors for Infectious Disease Diagnosis

Glycan derivatives have been applied to constructing glycan biosensors for virus detection. For example, Hushegyi et al. developed an impedimetric glycan biosensor for detecting lectins and influenza hemagglutinins (HAs) down to attomolar concentrations (aM). The biosensor was assembled onto modified gold electrodes with a mixed self-assembled monolayer of 11-mercaptoundecanoic acid (MUA) and 6-mercaptohexanol (MCH). Next, an amine-terminated glycan was coupled through EDC/NHS chemistry to form an amide covalent bond. As a result, a wide linear concentration range was obtained from attomolar to nanomolar for lectins and influenza HAs [112]. Similarly, Hushegyi et al. increased the selectivity of H3N2 influenza virus detection by the glycobiosensor using surface chemistry based on a mixed SAM composed of thiols bearing oligoethylene glycol (OEG) moieties resisting nonspecific interactions. The glycobiosensor was applied to detect H3N2 viruses, achieving a LOD of 13 viral particles in 1 μL, and was highly selective, enabling differentiation of H3N2 from the H7N7 influenza viruses [113]. Recently, *Soto* et al. developed an impedimetric peptide-based biosensor for detecting Spike protein, a SARS-CoV-2 envelope glycoprotein. The electrochemical biosensor was based on gold electrodes modified with a synthetic thiolated peptide bonded to Spike glycoprotein. The biosensor demonstrated a linear response of 0.05–1 μg mL^−1^ and a LOD of 18 ng mL^−1^. The biosensor could differentiate between positive and negative nasopharyngeal swab samples concerning the controls and differentiate the viral loading in clinical samples [39]. Santos et al. developed a capacitive biosensor to detect nonstructural glycoprotein 1 (NS1) related to dengue virus infection. The capacitive biosensor consisted of gold electrodes modified with a mixed SAM composed of 11-(ferrocenyl)-undecanethiol (11Fc) and polyethylene glycol-thiol (PEG-SH). An antibody was used as a biorecognition element to capture the NS1 glycoprotein on the electrode surface. The capacitive biosensor detected the molecular recognition event by a decrease in redox capacitance (C_r_) in a linear range from 1 to 5000 ng mL^−1^ and with a LOD of 340 pg mL^−1^ [125].

In the case of bacteria detection, Ma et al. reported polythiophene (PTPh) interface containing fused quinone moieties, which were then mannosylated to form a carbohydrate platform for *E. coli* detection. The bacteria detection was developed using Pili-Man and lipopolysaccharide (LPS)-ConA-Man binding approaches, being more sensitive than just the LPS-ConA-Man approach. Pili are multi-protein structures with adhesive properties related to the infectious ability of *E. coli* and LPS is the major component of the outer layer of the outer membrane of Gram-negative bacteria, such as *E. coli* and *Salmonella typhimurium* [126,127]. The electrochemical technique used to transduce the biorecognition event was SWV and demonstrated a LOD of 25 cells mL^−1^ and better selectivity and stability compared to the presently available technologies [128].

**Figure 3:**
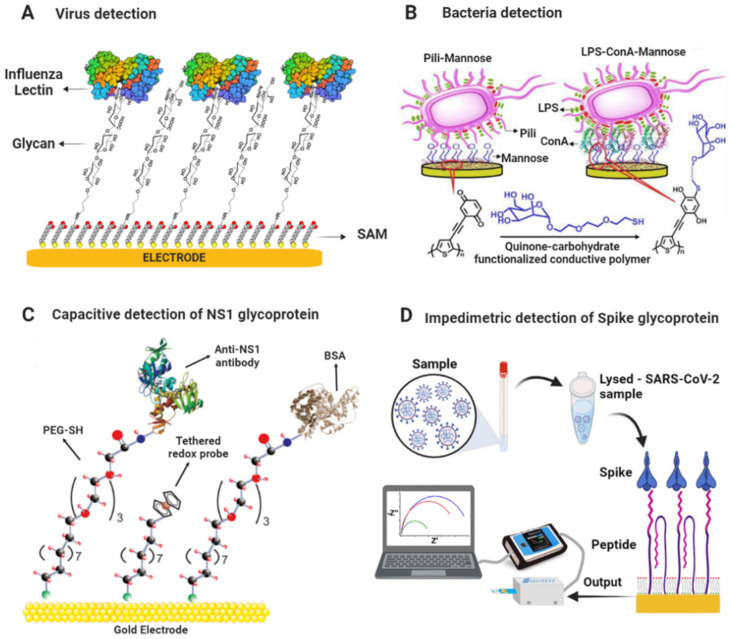
Scheme of glycobiosensors for pathogen detection. (**A**) Glycan-based biosensor for the detection of Influenza lectin [112]. (**B**) Biosensor based on redox-active conductive glycopolymer for *E. coli* detection. Adapted with permission from [128]. Copyright 2015 American Chemical Society. (**C**) Scheme of capacitive biosensing platform for detecting NS1 glycoprotein. Adapted from [125] with permission. Copyright Elsevier 2018. (**D**) Scheme of impedimetric biosensing platform for detecting Spike glycoprotein of SARS-CoV-2. Reproduced from [39] with permission. Copyright Elsevier 2022.

Cui et al. developed a label-free impedimetric glycobiosensor for quantitatively assessing interactions between pathogenic bacteria and mannose (Man). The sensing platform was based on gold electrodes modified with a SAM of Man/MUA/MCH and was applied to capture *E. coli* and *Salmonella typhimurium* bacteria. The sensing surface had a better binding affinity for *S. typhimurium* in a linear range of 50–1000 CFU mL^−1^ and LOD of 50 CFU mL^−1^ [129]. Similarly, Dechtrirat et al. detected GBPs and *E. coli* using an electrochemical displacement sensor based on ferrocene boronic acid as an electroactive reporter molecule and immobilized glycan. The sensor was based on gold electrodes modified with a SAM of thiolated-Man/OE conjugate and a ferrocene boronic acid (FcBA) pre-assembled as a reporter molecule onto the mannose surface. Upon the binding of GBP to the Man, the reporter molecule was displaced, and the decrease in SWV signal could be correlated to the GBP concentration. The sensor detected *E. coli* in a linear range from 6 × 10^2^ to 6 × 10^5^ cells mL^−1^ with a LOD of 6 × 10^2^ cells mL^−1^. In addition, it was highlighted that the sensor could complete a rapid analysis within 15 min [115]. Figure 3 shows a scheme of biosensing platforms for detecting viruses and bacteria mentioned above.

### 4.2. Glycobiosensors for Cancer Biomarker Detection

#### 4.2.1. CEA and CA 19-9 Glycoproteins

**Figure 4:**
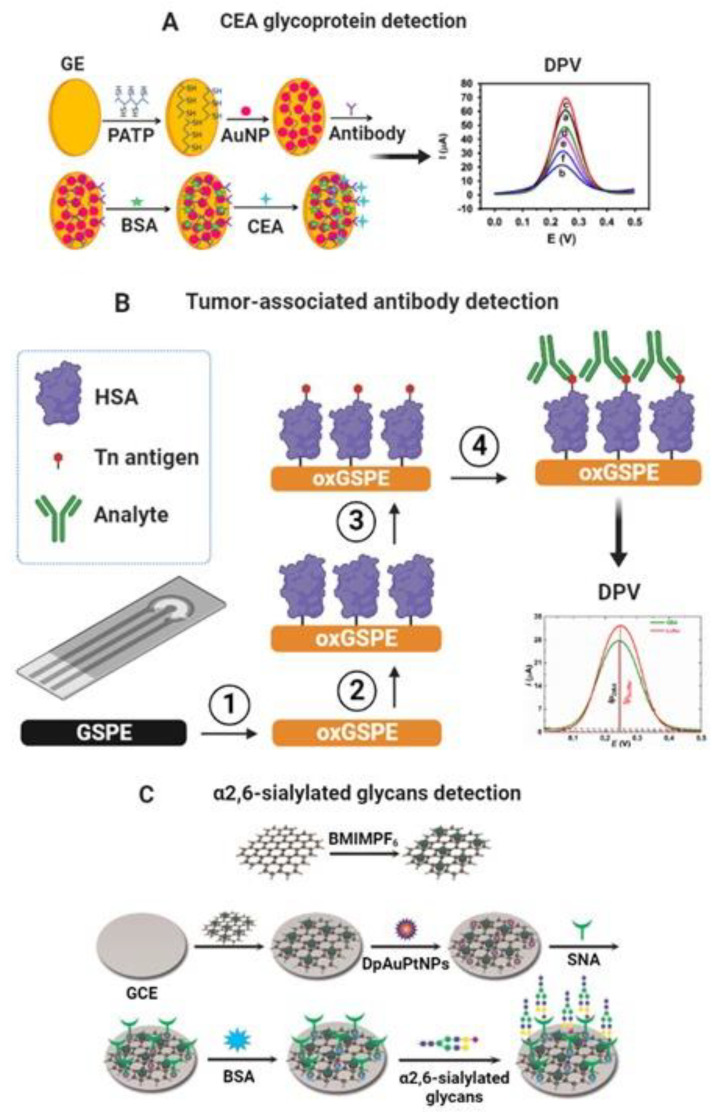
(**A**) Scheme of the different steps involved in developing a label-free nanoimmunosensor for CEA detection. (a) Bare gold electrodes and modified with (b) PATP, (c) PATP–AuNPs, (d) PATP–AuNPs–anti-CEA, (e) PATP–AuNPs–anti-CEA blocked with BSA, and (f) PATP–AuNPs–anti-CEA incubated with CEA after being blocked with BSA. Adapted from [98] with permission. Copyright Elsevier 2013. (**B**) Modification of graphene screen-printed electrode (GSPE) by electrochemical oxidation (step 1), covalent immobilization of human serum albumin (HSA) as a natural nanoscaffold (step 2), and covalent immobilization of a Tn antigen to HSA (step 3). The final step is incubation with the analyte protein (step 4) [135]. (**C**) Schematic representation of the electrochemical biosensor based on GCE modified with rGO-EPA/BMIMPF_6_/AuPtNPs. Reproduced from [142] with permission. Copyright Elsevier 2015.

CEA and CA-19-9 glycoproteins are cancer biomarkers detected using electrochemical glycobiosensors via label-free and label-based approaches. For label-free detection of CEA glycoprotein, Liu et al. synthesized a conducting polymer (poly (2-amino thiophenol), PATP) with incorporated Au nanoparticles (AuNPs), in which they adsorbed anti-CEA antibody for the sensitive and label-free electrochemical detection of CEA. The recognition of CEA was characterized using DPV with a linear range from 1 fg mL^−1^ to 10 ng mL^−1^ and LOD of 0.015 fg mL^−1^. Moreover, the results of CEA detection in real serum samples were consistent with those determined by a conventional immunoassay, showing the practical utility of the biosensor [98]. Likewise, Zhao et al. developed an electrochemical biosensing platform to detect CEA that employed lectin as a bioreceptor anchored at AuNPs and enzymatic catalysis for signal amplification. The biosensor was made up of electrodeposition of AuNPs on screen-printed carbon electrodes and self-assembly of cysteamine (Cys) on top of their surface for subsequent covalent coupling of the lectins via the amidation reaction. The target protein was then captured by the lectin-modified platform and made to react with a horseradish peroxidase (HRP) labeled anti-CEA antibody bioconjugate in a sandwich-type assay. The transduction of this molecular recognition event was made via chronoamperometry in the presence of hydroquinone (HQ) and hydrogen peroxide, achieving a linear concentration range of CEA from 0.5 ng mL^−1^ to 7 ng mL^−1^, with LOD of 0.01 ng mL^−1^ [130]. Figure 4A shows a schematic of the biosensing platform for detecting CEA glycoprotein.

Other researchers developed a polyaniline (PA) electrochemical derivative, a poly-(N,N’-diphenyl-p-phenylenediamine)-Au/Pt highly electroactive nanocomposite, as a platform to link the anti-CA 19-9 antibody for the label-free and sensitive detection of CA 19-9. The analytical performance of this immunosensor was tested via the SWV technique thanks to its intrinsic electrocatalytic activity, with H_2_O_2_ for signal recording. A wide linear range was obtained from 0.001 to 40 U mL^−1^ and ultralow LOD of 2.3x10^−4^ U mL^−1^. In addition, the biosensor was used to analyze clinical serum samples. The results agreed with those from the ELISA standard analysis, suggesting the potential application of this immunosensor for the clinical diagnosis of this biomarker [131]. Furthermore, Thapa et al. developed a highly sensitive capacitive biosensor to detect CA 19-9 glycoprotein based on gold electrodes modified with polyethyleneimine (PEI) and CNTs with an anti-CA 19-9 antibody immobilized covalently on the surface. The biosensor could detect CA 19-9 glycoprotein in a linear range of 0.05–60 U mL^−1^ with a LOD of 0.35 U mL^−1^ [132]. Figure 4A shows a schematic of the biosensing platform for detecting CEA glycoprotein.

#### 4.2.2. Protein Glycosylation

##### STn Antigen and Anti-STn Antibodies

The protein glycosylation can be altered in many diseases, including cancer. An example is the mucin-type O-glycoproteins that express the truncated glycans Thomsen-nouvelle (Tn) and sialyl-Tn (STn) [133]. Silva et al. developed an impedimetric biosensor based on the SNA I lectin immobilized on 16-mercaptohexadecanoic acid (MHDA) SAM-modified-gold electrodes to detect cancer-associated STn antigen. Lectin biosensor could detect transferrin, an STn-containing glycoprotein, in a linear range from 20 to 70 ng and LOD of 20 ng. The biosensor could detect the glycan from serum glycoproteins of cancer patients, and the complete assay took about 10 min [134]. Furthermore, Kveton et al. developed a glycobiosensor for detecting a Tn-associated antibody. The biosensor was built on an electrochemically activated/oxidized graphene screen-printed electrode (GSPE) for covalent attachment of human serum albumin (HSA). HSA acted as a natural nanoscaffold for covalent immobilization of Tn antigen to be fully available for affinity interaction with a tumor-associated antibody. The molecular binding of antibody and Tn antigen was monitored via DPV, achieving a linear range of 10 aM–10 pM and a LOD of 10 aM [135]. Figure 4B shows a schematic of the biosensing platform for detecting a tumor-associated antibody.

##### α2,3-Sialylated Glycans

Alteration of α2,3-sialylation is related to the development of certain cancers, in which sialylated glycoproteins can be released into the blood during apoptosis and detected circulating in serum [136]. There have been reports of some biosensors for detecting sialylated glycans useful for cancer diagnosis and clinical research. For example, Niu et al. used polyamidoamine (PAMAM) dendrimer conjugated with carboxyl-functionalized multiwalled carbon nanotubes (c-MWCNTs) for sensitive detecting of α2,3-, and α2,6-sialylated glycans in serum via DPV. 1,4-phenylene diisothiocyanate (PDITC) was used as a green homobifunctional cross-linker for SNA I and MAL lectin immobilization. Under optimal detection conditions, the linear range for α2,3-sialylated glycans was 10 fg mL^−1^- 50 ng mL^−1^ and for α2,6-sialylated glycans 10 fg mL^−1^–50 ng mL^−1^, respectively. The LOD was 3 fg mL^−1^ for both biosensors [137]. Similarly, for the determination of α2,3-sialylated glycans in serum, Niu et al. developed a biosensor using a GCE modified with c-MWCNTs, a PAMAM dendrimer, and the MAL lectin immobilized by the PDITC linker. The biosensor had a linear range of 10 fg mL^−1^–50 ng mL^−1^ with a LOD of 3 fg mL^−1^, and serum recovery experiments demonstrated high precision (around 100%) [138].

Yuan et al. assembled a sandwich-type biosensor for detecting α2,3-sialylated glycans based on fullerene–palladium–platinum alloy (n-C_60_-PdPt) and 4-mercaptophenylboronic acid (4-MPBA) nanoparticle hybrids coupled with Au-polymethylene blue-MAL lectin (Au-PMB-MAL) signal amplification. The biosensor was fabricated via surface modification of GCE with amino-functionalized fullerene coupled with n-C_60_-PdPt nanocrystals. The n-C_60_ nanomaterial had a large surface area for the on-site reduction of bimetallic alloy nanoparticles and high electron transfer. 4-MPBA were immobilized on the n-C_60_-PdPt by chemisorption of the thiol group and captured the α2,3-sialylated glycans by coordinating the boron atom of 4-MPBA to the amide group of Neu5Ac in the glycan structure. The MAL-Au-PMB nanocomposites recognized the α2,3-sialylated glycans attached to the electrode surface, enabling signal amplification via DPV. The sandwich-type biosensor demonstrated a wide linear range of 10 fg mL^−1^–100 ng mL^−1^ and a LOD of 3 fg mL^−1^. Furthermore, the biosensor could detect α2,3-sialylated glycans in serum samples, indicating its potential use in clinical applications [139].

##### α2,6-Sialylated Glycans

In addition, the alteration of α2,6-sialylation is also related to cancer development. Apoptotic cells overexpress the α2,6-sialylated glycans, which are released into the blood and can be detected in human serum [140]. Gao et al. fabricated an ultrasensitive electrochemical biosensor based on graphite oxide (GO), Prussian blue (PrB), and PTC-NH_2_ (an ammonolysis product of 3,4,9,10-perylenetetracarboxylic dianhydride) nanocomposite for the selective detection of α2,6-sialylated glycans. Glassy carbon electrodes (GCE) were modified with the nanocomposite and adsorbed AuNPs. An SNA I lectin was covalently immobilized onto the AuNPs, which detected α2,6-sialylated glycans. The glycobiosensor was applied to detect α2,6-sialylated glycans via DPV in human serum, and it worked well over a broad linear range (0.1 pg mL^−1–^500 ng mL^−1^) with a LOD of 0.03 pg mL^−1^ [141]. Afterward, Li et al. enhanced the SNA I lectin biosensor sensitivity toward detection in serum of α2,6-sialylated glycans. The improved glycobiosensor was based on GCE modified with a nanocomposite of reduced graphene oxide-tetraethylene pentamine (rGO-TEPA) and 1-butyl-3-methylimidazolium hexafluorophosphate (BMIMPF_6_). Bimetallic gold platinum alloy nanoparticles (AuPtNPs) were adsorbed on the nanocomposite surface, providing a large surface area for the immobilization of SNA I lectin. The lectin–glycan molecular biorecognition event was detected and quantified via amperometry in a linear range of 10 fg mL^−1^–1 μg mL^−1^ and a LOD of 3 fg mL^−1^ [142].

Niu et al. developed an ultrasensitive dual-type responsive electrochemical biosensor for detecting α2,6-sialylated glycans based on gold nanorods functionalized with streptavidin (AuNRs-SA) and SNA-biotinylated lectin that recognized the glycan and enabled detection via DPV. Carboxylated single-walled carbon nanohorns/sulfur-doped platinum nanocluster (c-SWCNHs/S-PtNC) was used as a signal label, showing excellent catalytic performance and allowing for the expansion of the ultrasensitive detection of α2,6-sialylated glycans. The c-SWCNHs/S-PtNC was functionalized with phenylboronic acid bound to the glycan–lectin complex in a sandwich-type format and significantly amplified the electrochemical signal recorded by amperometry. The sandwich-type biosensor possessed a wide linear range from 1 fg mL^−1^ to 100 ng mL^−1^ with a LOD of 0.69 fg mL^−1^. Furthermore, the biosensor exhibited excellent stability and recovery in serum samples, indicating its potential for use in clinical applications [143]. Zhao et al. developed a signal amplification system for the sensitive determination of α2,6-sialylated glycans in serum based on gold electrodes modified with SNA I lectin, which captured the α2,6-sialylated glycans and Fe-based metal–organic frameworks (Fe-MOFs) decorated with silver nanoparticles (AgNPs). Ag/Fe-MOFs nanocomposite was functionalized with SH-PEG-COOH to bind with 3-aminophenylboronic acid (M-APBA) via an amide bond, which recognized the α2,6-sialylated glycans. Ag/Fe-MOFs nanocomposite exhibited excellent redox properties enabling amplification signal via DPV; the linear range was 1 fg mL^−1^–1 ng mL^−1^ with a LOD of 0.09 fg mL^−1^ [144]. Figure 4C shows a schematic of the biosensing platform for detecting α2,6-sialylated glycans.

##### Glycosyltransferases

**Figure 5:**
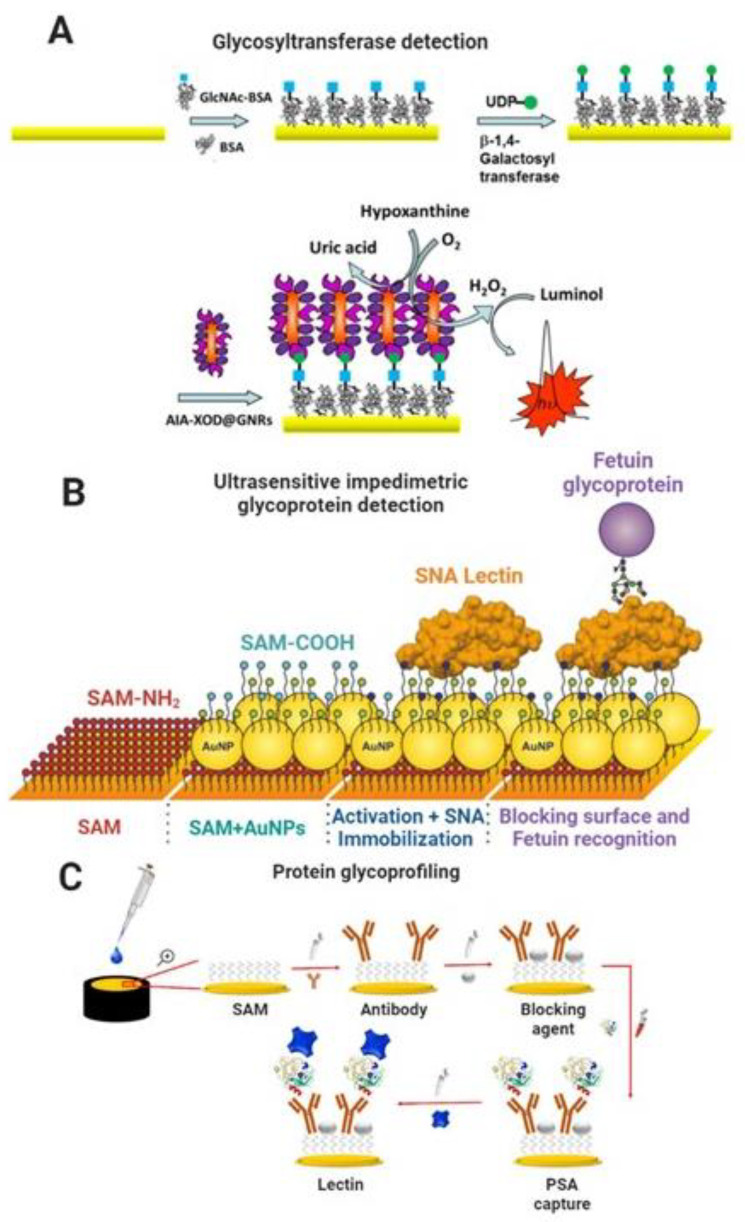
(**A**) Scheme of fabrication steps of biosensor based on a gold electrode modified with a bioconjugate of GlcNAc-BSA. Galactose transferred from UDP-Gal was specifically recognized by *Artocarpus integrifolia* lectin (AIA) immobilized on gold nanorods (GNRs) conjugated to xanthine oxidase (XOD). Reproduced from [147] with permission. Copyright Elsevier 2016. (**B**) Scheme of biosensor construction steps (from left to right): formation of a linker layer (NH_2_-terminated alkanethiol-AT) on a gold surface (1st SAM on AuE); deposition of gold nanoparticles (AuNPs) and formation of a 2nd mixed SAM layer consisting of 11-mercaptoundecanoic acid and 6-mercaptohexanol on AuNPs; activation of the carboxyl group, subsequent covalent attachment of SNA I lectin, and finally an application of the lectin biosensor in the biorecognition of a glycoprotein, fetuin (FET). Adapted from [152] with permission. Copyright Elsevier 2013. (**C**) Scheme of construction steps of the biosensor for detecting PSA and glycoprofiling of PSA by application of lectin. Reproduced from [155] with permission. Copyright Elsevier 2013.

Glycosyltransferases (GTs) are the enzymes that glycosylate proteins and other molecules. Specific GTs are overexpressed during the tumorigenesis process, making them suitable biomarkers for diagnosis/prognosis [145]. Biosensors based on electrochemiluminescence (ECL) for detecting glycosyltransferases have been published. Xie et al. developed a biosensor to analyze β-1,4-galactosyltransferase (Gal-T) activity based on a graphitic carbon nitride (g-C_3_N_4_) and polystyrene microsphere nanoprobe functionalized with a lectin. The strategy in the biosensor development involved conjugation of N-acetylglucosamine-BSA (GlcNAc-BSA) to g-C_3_N_4_ modified glassy carbon electrode, which exhibited a strong ECL signal. In the presence of Gal T and UDP-Gal as a co-substrate, galactose was transferred to the GlcNAc-BSA, and the ECL signal decreased slightly. Next, the signal decreased significantly by the lectin–galactose interaction because the nanoprobe’s poor conductivity inhibited the electron transfer at the electrode interface. The biosensor displayed high sensitivity for Gal-T activity detection with a low LOD of 7 × 10^−5^ U mL^−1^ [146]. Another similar strategy was developed by Chen et al., who used the same format of GlcNAc-BSA immobilized on gold electrodes and galactose conjugation to GlcNAc-BSA by Gal-T. The galactose was then specifically recognized by *Artocarpus integrifolia* lectin immobilized on gold nanorods conjugated to xanthine oxidase. The LOD obtained was 9 × 10^−4^ U mL^−1^ [147]. Figure 5A shows a schematic of the biosensing platform for detecting β-1,4-galactosyltransferase.

### 4.3. Ultrasensitive Impedimetric and Capacitive Biosensors for the Detection of Glycan-Based Biomarkers

Impedimetric and capacitive biosensors are among the most sensitive label-free analytical devices available [148,149]. In general, impedimetric and capacitive biosensors are sensitive to femtomolar to picomolar concentrations of the molecular target, and some publications have reported limits significantly below this [149,150,151]. Bertok et al. developed an ultrasensitive impedimetric glycobiosensor capable of detecting glycoproteins down to the aM level. The biosensing platform was assembled on aminoalkanethiol (SH-(CH_2_)_11_-NH_2_)/AuNPs-modified gold electrodes, and a lectin from *Sambucus nigra* (SNA I) was covalently immobilized onto the nanostructured surface. After that, the nonspecific binding sites were blocked with polyvinyl alcohol (PVA). Finally, the glycobiosensor was applied to detect glycoproteins fetuin and asialofetuin containing sialic acid. The linear range was from 1 aM to 10 pM and a LOD of 1 aM (e.g., 24 glycoprotein molecules in 40 µL of a sample or 40 yoctomoles) [152]. In addition, Bertok et al. covalently immobilized an SNA I lectin on a mixed-SAM of MUA and betaine-terminated thiol to resist nonspecific interactions. The glycobiosensor detected the fetuin glycoprotein within a linear range from 100 fM to 100 nM and a LOD of 100 fM [153]. Figure 5B shows a schematic of the ultrasensitive biosensing platform for detecting fetuin glycoprotein.

Pihíková et al. developed an ultrasensitive impedimetric biosensor for glycoprofiling PSA. The biosensor was based on gold electrodes modified with a SAM of MUA/MCH and an anti-PSA antibody covalently immobilized on the surface. After the antibody–antigen binding event, the biosensor was incubated with the lectin (SNA I) that recognized the glycan part of PSA (α-2,6 linked sialic acid). The impedimetric biosensor responded linearly from 3.4 aM to 380 pM with a LOD of 4 aM [154]. In addition, Pihíková et al. assembled the glycobiosensor on a SAM of MUA/MCH, optimized a blocking agent (carbo-free commercial solution), and glycoprofiled PSA with *Maackia amurensis* lectin (MAL, recognizing α-2,3-sialic acid) in serum samples. As a result, the glycobiosensor could detect PSA glycoprotein in serum in a linear range of 100 ag mL^−1^ to 1 µg mL^−1^ with a LOD of 100 ag mL^−1^ and discriminated serum samples from healthy individuals of prostate cancer patients [155]. Figure 5C shows a schematic of the biosensing platform for PSA protein and its glycoprofiling.

Chocholova et al. developed another glycoprofiling platform using an antifouling zwitterionic layer-based impedimetric biosensor for determining human epidermal growth factor receptor (HER2) in human serum. The biosensor was assembled on screen-printed carbon electrodes modified with zwitterionic hydrogels that resisted nonspecific protein adsorption and allowed covalent attachment of an anti-HER2 antibody. The biosensor could detect the analyte via EIS in a linear range from 0.1 to 10 ng mL^−1^ with a LOD of 5 pg mL^−1^ (77 fM). In addition, the results demonstrated a significant difference between a high-risk woman without breast cancer (BCa) vs. a woman with the second stage of BCa via glycan profiling using erythroagglutinin lectin from *Phaseolus vulgaris*, specific for complex and branched structures of GlcNAc linked to mannose [156].

Capacitive biosensors offer novelty applications to detect glycoproteins related to viruses and cancer biomarkers [149]. Wang et al. developed a capacitive biosensor using microwires coated with Zika or Chikungunya virus envelope antigen. The biosensor could detect ten antibody molecules in a 30 µL volume and could be used to determine the isotypes present in a serum sample [157]. Oliveira et al. developed a capacitive nanobiosensing interface to detect interleukin-6 (IL-6) glycoprotein. The capacitive biosensor consisted of carbon electrodes nanostructured with graphene oxide (GO) and the Prussian blue (PrB) redox-active compound. The capacitive nanobiosensor detected the molecular recognition event between an antibody and IL-6 by a decrease in C_r_ in a linear range from 0.2 ng mL^−1^ to 20 μg mL^−1^ and a LOD of 5.6 ng mL^−1^ [158]. Table 3 shows the summary of electrochemical glycobiosensors mentioned above.

In summary, electrochemical glycobiosensing is a versatile tool for detecting multiple disease biomarkers, as summarized in Table 3. Electrochemical glycobiosensors meet the REASSURED criteria (real-time connectivity, ease of specimen collection, affordable, sensitive, specific, user friendly, rapid, equipment free, and delivered to those who need it); these characteristics make them suitable devices for the point-of-care molecular diagnosis/prognosis of diseases [159]. Overall, voltammetry- and amperometry-based biosensors have advantages such as fast response, ease of use, capability for analyzing two or more analytes simultaneously in the same sample, and more straightforward data analysis. The main shortcoming of voltammetry- and amperometry-based biosensors is relatively low sensitivity and limited precision [160]. Instead, impedance- and capacitance-based biosensors have the main advantage of high sensitivity, and they avoid the need for modification of analyte recognition elements with redox mediators. The main shortcoming of impedance- and capacitance-based biosensors is that data analysis requires extensive knowledge of electrochemistry, and measurements are difficult to perform in portable systems [150]. The reproducibility of these electrochemical glycobiosensors is always a significant limitation. One way to handle this shortcoming is to properly design the biosensing surface to ensure a similar initial signal. This could be achieved by adjusting surface roughness, immobilizing a maximal quantity of bioreceptors on nanostructures, controlling the thickness of the detection film, and using stable nanomaterials and bioreceptors [14,15,161,162].

These shortcomings could be addressed by combining effective point-of-care electrochemical biosensing methods with intelligent software and data processing methods that enable global monitoring of diseases and real-time decision-making [22].

**Table 3:** Glycobiosensors for the detection of pathogens and cancer biomarkers reported in the last ten years.

Glycobiosensor Purpose	Sensing Platform Bioreceptor/Detection Technique	Target	Limit of Detection (LOD)	Linear Range	Ref.
Pathogens	SAM (MUA/MCH) Glycan-NH_2_/EIS	HAs	8 aM	8 aM–80 nM	[112]
	SAM (OEG/OEG-COOH) Glycan-NH_2_/EIS	H3N2	13 viral particles µL^−1^	10–100^3^ viral particles µL^−1^	[113]
	SAM (Peptide) Peptide/EIS	SARS-CoV-2	18 ng mL^−1^	0.05–1 µg mL^−1^	[39]
	SAM (PEG-SH/11Fc) Antibody/ECS	NS1	340 pg mL^−1^	1–5000 ng mL^−1^	[125]
	Microwires/SAM Envelope Protein E/capacitance	Antibodies anti-ZIKV E	10 antibodies in 30 µL	10–10^3^ antibodies in 30 µL	[157]
	SAM (MUA/MCH) Mannose/EIS	*S. typhimurium*	50 CFU mL^−1^	50–10^3^ CFU mL^−1^	[129]
	PTPh-quinone Mannose/SWV	*E. coli*	25 cells mL^−1^	2 × 10^3^–5 × 10^4^ cells mL^−1^	[128]
	SAM (OEG/FcBA) Mannose/SWV		6 × 10^2^ cells mL^−1^	6 × 10^2^–6 × 10^5^ cells mL^−1^	[115]
Cancer	PATP/AuNPs Antibody/DPV	CEA	0.015 fg mL^−1^	1 fg mL^−1^–10 ng mL^−1^	[98]
	AuNPs/Cys Lectin/chronoamperometry		0.01 ng mL^−1^	0.5–7 ng mL^−1^	[130]
	PA/Au-Pt Antibody/SWV	CA-19-9	2 × 10^−4^ U mL^−1^	0.001–40 U mL^−1^	[131]
	PEI/CNTs Antibody/EIS		0.35 U mL^−1^	0.05–60 U mL^−1^	[132]
	SAM (MHDA) Lectin/EIS	STn	20 ng	20–70 ng	[134]
	GSPE/HSA Tn antigen/DPV	Antibody anti-Tn	10 aM	10 aM–10 pM	[135]
	PAMAM/c-MWCNTs/PDITC Lectin/DPV	α2,3-sialylated glycans	3 fg mL^−1^	10 fg mL^−1^–50 ng mL^−1^	[137]
	n-C_60_-PdPt/4-MBPA/Au-PMB Lectin/DPV		3 fg mL^−1^	10 fg mL^−1^–100 ng mL^−1^	[139]
	GO-PrB-PTC-NH_2_/AuNPs Lectin/DPV	α2,6-sialylated glycans	0.03 pg mL^−1^	0.1 pg mL^−1^–500 ng mL^−1^	[141]
	rGO-TEPA-BMIMPF_6_/AuPtNPs Lectin/amperometry		3 fg mL^−1^	10 fg mL^−1^–1 µg mL^−1^	[142]
	AuNRs-SA/c-SWCNHs/S-PtNC Lectin/amperometry		0.69 fg mL^−1^	1 fg mL^−1^–100 ng mL^−1^	[143]
	Ag/Fe-MOFs/M-APBA Lectin/DPV		0.09 fg mL^−1^	1 fg mL^−1^–1 ng mL^−1^	[144]
	g-C_3_N_4_/GlcNAc-BSA Lectin/ECL	β-1,4-GalT	7 × 10^−5^ U mL^−1^	5 × 10^−4^–0.05 U mL^−1^	[146]
	GlcNAc-BSA Lectin/ECL		9 × 10^−4^ U mL^−1^	0.001–0.1 U mL^−1^	[147]
	SAM (SH-(CH_2_)_11_-NH_2_)/AuNPs) Lectin/EIS	Fetuin	1 aM	1 aM–10 pM	[152]
	SAM (MUA/betaine) Lectin/EIS		100 fM	100 fM–100 nM	[153]
	SAM (MUA/MCH) Antibody/EIS	PSA	4 aM	3.4 aM–380 pM	[154]
	SAM (MUA/MCH/carbo-free) Antibody/EIS		100 ag mL^−1^	100 ag mL^−1^–1 µg mL^−1^	[155]
	Zwitterionic hydrogel Antibody/EIS	HER2	5 pg mL^−1^	100 pg mL^−1^–10 ng mL^−1^	[156]
	GO/PrB Antibody/ECS	IL-6	5.6 ng mL^−1^	0.2 ng mL^−1^–20 µg mL^−1^	[158]

## 5. Concluding Remarks, Current Challenges and Opportunities

Glycans are biomolecules with relevant biological functions. They participate during infections provoked by pathogens and cellular expression changes related to more complex processes, particularly during cancer development [5,7]. Glycans are structurally diverse and complex. However, glycosylation is typically site specific, and specific types of glycans are present on restricted subsets of glycoproteins [145]. All of these indicate that glycans are helpful as disease biomarkers because cells express specific types of glycoproteins and release them into body fluids during disease progression. Furthermore, glycans are functional as biorecognition elements and can be easily incorporated into electrochemical biosensors to detect multiple analytes. The synergy of glycans as highly specific bioreceptors and proper transduction techniques in electrochemical biosensors enables the sensitive and specific detection of multiple molecular targets. Furthermore, incorporating nanomaterials into the electrode surface may improve the biosensor’s analytical performance because these nanostructures have a large surface area for bioreceptor immobilization and can promote a fast electron transfer.

There are different approaches to immobilizing the biomolecules on the electrode, depending on the chemical composition of the biomolecules and electrode surface. The biofunctionalization process is characterized by different physicochemical techniques to confirm successful biosensor assembly. In particular, the biofunctionalization process and the bioreceptor–analyte molecular biorecognition event can be monitored using highly sensitive electrochemical techniques. Electrochemical glycobiosensors can perform similarly or better than conventional clinical methods and offer a practical approach to detecting different molecular targets. Electrochemical glycobiosensors have high sensitivity, specificity, selectivity, rapid response, user-friendly, and cost-effective features, as well as the possibility of miniaturization to deliver devices at the point of care.

Glycan-based electrochemical biosensors still have some weaknesses and limitations. A challenge to overcome in the electrochemical glycobiosensor field is that glycans are biomolecules very sensitive to environmental conditions and can rapidly lose their biological activity over time. For this reason, it is mandatory to incorporate glycan-based biorecognition elements with improved long-term stability into electrochemical biosensors that allow the development of efficient, robust, and low-cost analytical devices delivered to the end user ready to use.

Yet, glycan-based electrochemical biosensors offer tremendous opportunities for ultrasensitive biomarker monitoring at the POC. For example, developing new nanomaterials with improved electrochemical performance and new surface chemical moieties allows convenient and efficient biomolecule immobilization. Furthermore, electrochemical glycobiosensors can be incorporated into microfluidic platforms to develop fast detection assays with minimal sample manipulation by the user. This approach could also detect different molecular targets in a multiplexed format. Multiple working electrodes are individually modified in multiplexing formats with different bioreceptors to detect various analytes simultaneously. This approach enables the determination of multiple levels of molecular markers (e.g., nucleic acids, proteins, and metabolites), paving the way for precision medicine and providing a detailed disease characterization to customize healthcare [3]. Furthermore, the signal can be acquired using portable systems, such as hand potentiostats with a smartphone’s signal readout. These attributes pave the way to personalized medicine enabling diagnosis/prognosis of diseases in decentralized settings, at the POC, closer to the patient.

The state of the art reviewed here demonstrates that ultrasensitive glycan-based nanobiosensing interfaces could be promising approaches to detect and quantify molecular targets in body fluids, thus holding considerable potential for determining cancer biomarkers and other infectious diseases in decentralized settings with a minimal reagent consumption and user-friendly operation mode.

## Figures and Tables

**Table 1:** Table of electrochemical glycobiosensors and analytical performance.

Electrochemical Technique	Sensing Principle	The Typical Range of the Limit of Detection (LOD)	Reference
CV	Application of a time-dependent potential to an electrochemical cell and measuring the resultant current as a function of the applied potential	10^−6^–10^−15^ M	[69]
DPV			
SWV			
Amperometry			
Potentiometry	Perturbation of the potential of the electrochemical cell	10^−3^–10^−6^ M	[59]
Conductometry	Quantification of the conductance change in the electrochemical cell		
EIS	Application of a small sinusoidal voltage perturbation in a range of frequencies while monitoring the resulting current	10^−9^–10^−18^ M	[22]
ECS			

Abbreviations: CV: cyclic voltammetry. DPV: differential pulse voltammetry. ECS: electrochemical capacitance spectroscopy. EIS: electrochemical capacitance spectroscopy. SWV: square wave voltammetry.

**Table 2:** Characterization techniques of electrochemical glycobiosensors.

Characterization Technique	Properties	Technique Principle	References
AFM	Morphology	Measurement of intermolecular forces and “seeing” atoms by using probe surfaces.	[70]
SEM/EDX	Morphology Composition	Application of kinetic energy to produce signals from the interaction of the electrons (secondary, backscattered, and diffracted backscattered). Secondary and backscattered electrons are used to visualize the morphology, and backscattered are related to composition.	[71,72]
FT-IR	Surface chemical composition	Measurement of the vibrations of atoms, and from this, functional groups are determined.	[73]
XPS	Surface chemical composition	The sample is irradiated with an X-ray, and some electrons become excited enough to escape from the atoms. The photo-ejected electrons are collected by an electron analyzer that measures their kinetic energy, allowing the element to be identified.	[73]
TGA, DSC	Sorption Composition	The sample is heated or cooled under controlled conditions and changes in some physical properties are measured.	[74,75]
UV-vis, PL	Optical	Light absorption and scattering by a sample.	[76]
CV, DPV, EIS, ECS	Electron transfer kinetics	Perturbation of the electrode by applying an electric potential and recording the resulting current.	[77,78]
Biacore	Bioreceptors affinity	The change in SPR response is measured after association/dissociation of a bioreceptor and ligand, respectively, with the sample flow in a microfluidic channel.	[80]

Abbreviations: AFM: atomic force microscopy. CV: cyclic voltammetry. DPV: differential pulse voltammetry. DSC: differential scanning calorimetry. ECS: electrochemical capacitance spectroscopy. EIS: electrochemical impedance spectroscopy. EDX: energy-dispersive X-ray spectroscopy. FT-IR: Fourier transform infrared spectroscopy. PL: photoluminescence. SEM: scanning electron microscopy. TGA: thermogravimetric analysis. UV-vis: ultraviolet-visible spectroscopy. XPS: X-ray photoelectron spectroscopy.

## Data Availability

Not applicable.
